# Outcomes of patients with hematologic malignancies and COVID-19 from the Hematologic Cancer Registry of India

**DOI:** 10.1038/s41408-021-00599-w

**Published:** 2022-01-05

**Authors:** Arihant Jain, Lingaraj Nayak, Uday Prakash Kulkarni, Nikita Mehra, Uday Yanamandra, Smita Kayal, Sharat Damodar, Joseph M. John, Prashant Mehta, Suvir Singh, Pritesh Munot, Sushil Selvarajan, Venkatraman Radhakrishnan, Deepesh Lad, Rajan Kapoor, Biswajit Dubashi, Ram S. Bharath, Hasmukh Jain, P. K. Jayachandran, Jeyaseelan Lakshmanan, Thenmozhi Mani, Jayashree Thorat, Satyaranjan Das, Omprakash Karunamurthy, Biju George, Manju Sengar, Pankaj Malhotra

**Affiliations:** 1 Department of Clinical Hematology and Medical Oncology, Postgraduate Institute of Medical Education and Research, Chandigarh, India; 2 Adult Haematolymphoid Disease Management Group, Tata Memorial Centre, Mumbai, India; 3 Department of Haematology, Christian Medical College, Vellore, India; 4 Department of Medical Oncology, Cancer Institute (WIA), Chennai, India; 5 Army Hospital (Research & Referral), New Delhi, India; 6 Department of Medical Oncology, Jawaharlal Institute of Postgraduate Medical Education & Research, Puducherry, India; 7 Department of Haematology, Mazumdar Shaw Medical Centre, Narayana Health City, Bangalore, India; 8 Department of Clinical Haematology-Oncology and Bone Marrow(Stem Cell) Transplantation, Christian Medical College, Ludhiana, India; 9 Department of Medical Oncology, Asian Institute, Faridabad, India; 10 Department of Clinical Haematology, Dayanand Medical College, Ludhiana, India; 11 Department of Biostatistics, Christian Medical College, Vellore, India

**Keywords:** Leukaemia, Infectious diseases

**Dear Editor**,

Several registry studies from high socio-demographic Index (SDI) countries have shown that COVID-19 illness in patients with hematological malignancies is associated with worse outcomes [1–6]. There are limited data regarding the outcomes of such patients from low SDI countries [7]. To understand the *real-world* outcome of COVID-19 patients with hematologic malignancies(HM) from India, the COVID-19 Hematologic Cancer Registry of India (CHCRI) started collecting data from patients of all ages with a current or history of HM and a laboratory-confirmed (positive RT-PCR or antigen test) COVID-19 infection from ten tertiary referral centers across India. These patients either attended the outdoor clinic or were admitted to these hospitals with COVID-19 illness. The current analysis describes the outcome of patients reported to the registry from March 21st, 2020, till March 20th, 2021. Data were submitted via individual case entry through an online CHCRI case record form maintained by Hematology Cancer Consortium(HCC). The cases from each site were not necessarily consecutive, and the denominator of cases at each site is not known. Data collection was retrospective from 21st March 2020 till 30th October 2020 and prospective from 1st November 2020 till 20th March 2021. The status of all patients was updated on May 1st, 2021. Double entries, nonhematologic diagnoses, and entries with incomplete outcome information were excluded from the analysis. The local Institutional Review Boards (IRB) approved the study at each center, and the study procedures complied with the Helsinki declaration. Any patient who received any form of therapy for the HM in the past 4 weeks was defined to be on active anticancer therapy. A delay in planned anticancer therapy by more than 2 weeks was considered as “interruption”, while the use of lower dose chemotherapy was considered “de-escalation”. COVID-19 severity was classified as mild: asymptomatic for COVID-19-related symptoms (asymptomatic positive) or SpO2 > 94% on room air; moderate: SpO2 ranging from 90 to 94% on room air; Severe: SpO2 < 90% on room air. The disease severity was analyzed using univariate and multivariable penalized logistic regression analysis. The Kaplan Meier method was used for time-to-event outcomes, and the Log-rank test was used to compare two survival curves. The variables significant at *p*-value < 0.05 were included in the multivariable Cox regression model.

Table 1 shows the baseline study characteristics and mortality among the three COVID-19 severity categories. Among the 565 patients of COVID-19 with HM reported to the registry over 12 months, 379 (67.1)% patients had mild COVID-19 while 71 (12.6%) and 115 (20.5%) patients had moderate and severe COVID-19, respectively. Seventy-six percent of these patients were admitted to the hospital (Supplementary Fig. 1, 2). The mean age of the whole cohort was 41 (SD±19) years, and the male: female ratio was 2.2:1. In the entire cohort, the three common diagnoses in order of prevalence were Acute Lymphoblastic Leukemia (ALL), lymphoma, and Multiple Myeloma. Most of the patients (66%), had COVID-19 within six months of being diagnosed with HM. 30% of patients were in remission for their HM at the time of COVID-19 diagnosis. 76% of patients were receiving active anticancer therapy when COVID-19 was diagnosed. 50.5% of patients received steroids, and 15.2% had received monoclonal antibodies in the previous 4 weeks as a part of their treatment for HM.

**Table 1:** Clinical characteristics and outcome of patients with Hematologic Malignancy and COVID-19.

Variables	All patients^a^	Overall and COVID-19 severity specific mortality^a^ [deaths/patients(%)]			
	(*N* = 565)	Overall (*n* = 565)	Mild (*n* = 379)	Moderate (*n* = 71)	Severe (*n* = 115)
*Age group (years)*					
≤20	94 (16.6)	15/94 (15.6)	9/81 (11.1)	2/7 (28.6)	4/6 (66.7)
21–40	183 (32.4)	32/183 (17.5)	9/129 (6.7)	2/20 (10)	21/34 (61.7)
41–60	188 (33.4)	38/188 (20.2)	7/119 (5.9)	3/26 (11.5)	28/43 (65.1)
>60	100 (17.7)	31/100 (31)	5/50 (10)	4/18 (22.2)	22/32 (68.8)
All patients	565	116/565 (20.5)	30/379 (7.9)	11/71 (15.5)	75/115 (65.2)
*Gender*					
Male	392 (69.4)	79/392 (20.1)	15/257 (5.8)	6/49 (12.2)	58/86 (67.4)
Female	173 (30.6)	37/173 (21.4)	15/122(12.3)	5/22 (22.7)	17/29 (58.6)
*Diabetes*	81 (14.3)	20/81 (24.7)	2/44 (4.6)	2/11 (18.2)	16/26 (61.5)
*Hypertension*	71 (12.6)	13/71 (18.3)	1/38 (2.6)	1/9 (11.1)	11/24 (45.8)
*Hematologic malignancy subtype*					
Acute lymphoblastic leukemia	155 (27.4)	23/155(14.8)	7/123 (5.7)	1/12 (8.3)	15/20 (75)
Acute myeloid leukemia	77 (13.6)	32/77(41.6)	10/39 (25.6)	2/11 (18.2)	20/27 (74.1)
Non-Hodgkin lymphoma (High grade)	118 (20.9)	20/118 (17)	4/84 (4.8)	5/13 (38.5)	11/21 (52.4)
Non-Hodgkin lymphoma (Low grade)^b^	57 (10.1)	12/57 (21)	3/31 (9.7)	0/11 (0)	9/15 (60)
Hodgkin lymphoma	27 (4.8)	3/27 (11.1)	1/20 (5)	1/4 (25)	1/3 (33.3)
Multiple myeloma	93 (16.5)	20/93 (21.5)	3/53 (5.7)	2/16 (12.5)	15/24 (62.5)
Chronic myeloid leukemia	23 (4.1)	3/23 (13)	0/16 (0)	0/3 (0)	3/4 (75)
Others^c^	15 (2.7)	3/15 (20)	2/13 (15.4)	0/1 (0)	1/1 (100)
*Malignancy diagnosis to COVID-19 diagnosis interval* *≤* *6 months*	370 (65.5)	70/370 (18.9)	17/250 (6.8)	8/48 7)	45/72 (62.5)
*Malignancy in remission at the time of COVID-19 diagnosis*	149/552 (27)	22/149 (14.8)	7/112 (6.3)	1/16 (6.3)	14/21 (66.7)
*Systemic anticancer therapy at the time of COVID -19 diagnosis*	427/564 (75.7)	85/427 (20)	20/288 (6.9)	8/51 (15.7)	57/88 (64.8)
*Malignancy therapy Interruption/de-escalation*	398/563 (70.7)	97/398 (24.4)	20/251 (8)	8/47 (17.0)	69/100 (69)
*Decision forgoing ICU in favor of Palliation*	42/254 (16.5)	37/42 (88)	9/9 (100)	2/4 (50)	26/29 (89.7)
*Steroids in previous 4 weeks*	276/547 (50.5)	54/276 (19.6)	9/185 (4.9)	6/30 (20)	39/61 (63.9)
*Monoclonal antibodies in previous 4 weeks*^*d*^	86 (15.2)	15/86 (17.5)	3/62 (4.8)	3/8 (37.5)	9/16 (56.3)
*Post-transplant (n* *=* *26)*	26 (4.6)	11/26 (42.3)	4/15 (26.7)	1/3 (33.3)	6/8 (75)
Autologous-HCT	19 (73.1)	8/19 (42.1)	3/11 (27.2)	1/2 (50)	4/6 (66.7)
Allogeneic-HCT	7 (26.9)	3/7 (42.9)	1/4 (25)	0/1 (0)	2/2 (100)
*Laboratory parameters at COVID-19 diagnosis*					
Absolute neutrophil count <0.5 ×10^9^/L	86/375 (22.9)	27/ 86(31.4)	4/51 (7.8)	4/13 (30.8)	19/22 (86.4)
Absolute lymphocyte count <0.5 ×10^9^/L	134/371 (36.1)	31/134 (23.1)	2/80 (2.5)	5/20 (25)	24/34 (70.6)
D-dimer >2000 ng/ml	55/235 (23.4)	23/55 (41.8)	2/24 (8.3)	2/8 (25)	19/23 (82.6)
C-Reactive Protein >20 mg/L	98/224(43.8)	28/98(28.6)	2/45 (4.4)	1/18 (5.6)	25/35 (71.4)
Ferritin ≥ 500 ng/ml	171/231 (74)	51/171 (29.8)	5/80 (6.3)	8/33 (24.2)	38/58 (65.5)
*COVID-19 specific treatment*					
Steroid	243/558 (43.6)	70/243 (28.8)	7/102 (6.9)	6/52 (11.5)	57/89(64)
Remdesivir	114/555 (20.5)	42/114 (36.8)	3/28 (10.7)	4/29 (13.8)	35/57(61.4)
Favipiravir	17/555 (3.1)	2/17 (11.8)	1/12 (8.3)	0/3 (0)	1/2 (50)
Hydroxychloroquine	11/555 (2)	2/11 (18.1)	0/5 (0)	0/2 (0)	2/4 (50)
Tocilizumab	15/553 (2.7)	8/15 (53.3)	0/1 (0)	1/2 (50)	7/12 (58.3)
Prophylactic anticoagulant	124/555 (22.3)	36/124 (29)	1/41 (2.4)	5/33 (15.2)	30/50 (60)
Therapeutic anticoagulant	26/555 (4.7)	9/26 (34.5)	0/8 (0)	0/4 (0)	9/14 (64.3)
Convalescent plasma	3/556 (0.5)	2/3 (66.7)	0/1 (0)	0/0	2/2 (100)
High flow nasal canula/ventilatory support	86/556 (15.5)	58/86 (67.4)	0/0	6/16 (37.5)	52/70 (74.3)

^a^Cases were included for an outcome only if information of that outcome was reported.

^b^Includes chronic lymphocytic leukemia.

^c^Includes Myelodysplastic Syndrome, Philadelphia negative myeloproliferative neoplasms and plasma cell dyscrasias other than multiple myeloma.

^d^Includes Rituximab, Daratumumab and Nivolumab.

The diagnosis of COVID-19 had a significant impact on anticancer therapy, which was interrupted and or de-escalated in 71% of cases. The treatment of COVID-19 consisted of antiviral remdesivir in 21%, steroids in 44%, and prophylactic anticoagulation in 22% of patients. Less than 5% of patients received favipiravir, hydroxychloroquine (HCQ), tocilizumab, therapeutic anticoagulation, or convalescent plasma. 15.5% of patients in the whole cohort received High flow nasal cannula (HFNC) and/or ventilatory support. Among the patients with moderate/severe COVID-19, none of the COVID-19 directed drugs were associated with decreased mortality (Supplementary Table 1).

In consultation with the patient and their family members, the treating physician decided to withhold intensive chemotherapy and continue palliative therapy in 16.5% of patients. At a median follow-up of 90 days (IQR 42–180), 116 patients (20.5%) expired, of which 75 (64.7%) patients had severe COVID-19. Among all the patients who did not survive, 60/116 (51.7%) expired within 14 days from the COVID-19 diagnosis. Twenty-six patients (4.6%) in the cohort had received a Hematopoietic stem cell transplant (HCT), of which 11 patients died. 54.5% (6/11) of post-HCT patients who died had a severe COVID-19 illness.

On univariate analysis, age >60 years (HR 2.26, 1.21–4.23), diagnosis of acute myeloid leukemia (HR 3.27, 1.89–5.68), anticancer therapy interruption/ de-escalation (HR 2.43, 1.46–4.02), post-HCT status (HR 2.83, 1.51–5.28), absolute neutrophil count (ANC) < 0.5 ×10^9^/L (HR 1.71, 1.05–2.77), plasma D-dimer > 2000 ng/ml (HR 3.06, 1.71–5.47), serum ferritin ≥ 500 ng/ml (HR 3.15, 1.35–7.35) were the factors associated with increased mortality. Age > 60 years (HR 2.55, 1.23–5.27), diagnosis of acute myeloid leukemia (HR 2.85, 1.58–5.13), post-HCT status (HR 3.68, 1.82–7.45), and anticancer therapy interruption or de-escalation (HR 2.78, 1.65–4.68) were the significant factors for mortality on multivariable analysis (Table 2, Supplementary Fig. 3). In contrast, increasing age [20–40 years (OR 2.60 (1.31–5.15), 40–60 years (OR 3.44, 1.60–7.41), more than 60 years (OR 5.70, 2.43–13.35)], acute myeloid leukemia (OR 2.73, 1.45–5.12), and malignancy not being in remission (OR 1.85, 1.18–2.89) were the factors that were significantly associated with risk of developing severe COVID-19 on multivariable analysis. Recent exposure to corticosteroids or monoclonal antibodies was not related to the risk of developing severe COVID-19 in the current cohort (Supplementary Table 2).

**Table 2:** Univariate and multivariable cox regression analysis of factors affecting mortality^a^.

Variables	Univariate model for mortality		Multivariable model for mortality	
	HR (95% CI)	*P*-Value	HR (95% CI)	*P*-Value
*Age*				
≤20	1.00		1.00	
21–40	1.09 (0.58–2.03)	0.796	1.01 (0.53–1.92)	0.973
41–60	1.38 (0.76–2.51)	0.296	1.50 (0.75–3.00)	0.255
>60	2.26 (1.21–4.23)	**0.011**	2.55 (1.23–5.27)	**0.012**
*Diabetes*	1.32 (0.8–2.16)	0.278	–	–
*Hypertension*	0.99 (0.55–1.77)	0.974	–	–
**Hematologic malignancy related factors**				
*Malignancy subtype*				
Acute lymphoblastic leukemia	1.00		1.00	
Acute myeloid leukemia	3.27 (1.89–5.68)	**<0.001**	2.85 (1.58–5.13)	**<0.001**
Non-Hodgkin lymphoma (low grade)^b^	1.22 (0.56–2.65)	0.613	0.87 (0.37–2.05)	0.746
Non-Hodgkin lymphoma (high grade)	1.15 (0.62–2.13)	0.651	0.78 (0.39–1.57)	0.489
Hodgkin lymphoma	0.78 (0.23–2.6)	0.684	0.51 (0.15–1.75)	0.286
Multiple myeloma	1.72 (0.94–3.15)	0.079	0.68 (0.31–1.48)	0.331
Chronic myeloid leukemia	0.92 (0.28–3.08)	0.894	0.97 (0.28–3.41)	0.960
Others^c^	1.47 (0.44–4.91)	0.533	1.19 (0.34–4.13)	0.786
*Malignancy diagnosis to COVID-19 diagnosis interval*				
<6 Months	1.00			
>6 Months	1.32 (0.9–1.94)	0.151	–	–
*Malignancy status*				
Not in remission	1.57 (0.98–2.51)	0.059	–	–
Remission	1.00			
*Malignancy therapy interruption or de-escalation*				
Yes	2.43 (1.46–4.02)	**0.001**	2.78 (1.65–4.68)	**<0.001**
No	1.00			
*Systemic anticancer therapy*				
Yes	0.93 (0.6–1.45)	0.750	–	–
No				
*Steroids (previous 4 weeks)*				
Yes	0.95 (0.64–1.39)	0.778	–	–
No	1.00			
*Monoclonal antibody in previous 4 weeks*^d^				
Yes	0.68 (0.37–1.23)	0.201	–	–
No	1.00			
*Status post stem cell transplant*				
Yes	2.83 (1.51–5.28)	**0.001**	3.68 (1.82–7.45)	**<0.001**
No	1.0			
**Laboratory parameters at COVID-19 diagnosis**				
*D-dimer(ng/mL)*				
<1000	1.00			
1000–2000	1.19 (0.49–2.91)	0.701	–	–
>2000	3.06 (1.71–5.47)	**<0.001**	–	–
*Ferritin(ng/ml)*				
<500	1.00			
≥500	3.15 (1.35–7.35)	**0.008**	–	–
*Absolute neutrophil count*				
≥0.5 ×10^9^/L	1.00			
<0.5 ×10^9^/L	1.71 (1.05–2.77)	**0.032**	–	–

^a^Cases were included for an outcome only if information of that outcome was reported.

^b^Includes chronic lymphocytic leukemia.

^c^Includes Myelodysplastic Syndromes, Philadelphia negative myeloproliferative neoplasms and plasma cell dyscrasias other than multiple myeloma.

^d^Includes Rituximab, Daratumumab and Nivolumab.

Bold values indicate statistical significance.

The study presents registry-based data of patients with HM and COVID-19 from one of the worst-hit low SDI countries in the initial 12 months. Several registry-based retrospective studies are describing the short-term follow-up (up to 6 weeks) outcomes of patients with HM and COVID-19 [5, 6, 8–11]. Most of these studies are from regions with well-equipped healthcare resources and predominantly include patients with severe COVID-19 (>70% in most studies). The mortality in hospitalized/severe COVID-19 in the high SDI countries exceeds 30% (Supplementary Table 3). We noted a mortality rate of 20.5% in our whole cohort that had 67% patients with mild COVID-19. The mortality was ~8%, 16%, and 65% in patients with mild, moderate, and severe COVID-19, respectively. Age > 60 years is a common factor affecting mortality in the current as well as other studies. However, concerning the risk of developing severe COVID-19, any age >20 years was found significant in the current study. Most studies concur that patients with Acute Myeloid Leukemia (AML), lymphoma, and Multiple Myeloma have an increased risk for COVID-19 mortality [6, 9, 11, 12]. In the current study, AML was associated with an increased risk of mortality, while low-grade lymphoma including Chronic Lymphocytic Leukemia and Multiple Myeloma were only associated with an increased risk of severe COVID-19. A quarter of AML patients died within a month of COVID-19 diagnosis; the risk of death in multiple myeloma patients was uniform over the six-month follow-up period (Supplementary Table 4). In the current study, the use of steroids or monoclonal antibodies as a part of treatment for underlying malignancy was not associated with either COVID-19 severity or mortality. These results have been contradictory in lymphoma patients in different studies [12, 13]. With regards to outcomes in post-HCT patients, our study concurs with the CIBMTR and EBMT studies, which also showed an increased risk of COVID-19 mortality post-HCT [14, 15].

Small patient numbers and prescriber biases in treatment and access may have contributed to the lack of association with COVID-19 specific therapies like antivirals, steroids, or anticoagulation and mortality. Two Spanish studies also have shown a contrary association of steroids with mortality [10, 11]. Frequently, patients with HM are already on steroids for their malignancy at the time of COVID-19 diagnosis; this may also confound outcomes.

The significant limitations of this study are referral bias, differential access to healthcare, heterogeneous treatment policies with the data source limited to tertiary referral centers. The true denominator of cases from a given site was not known. Though treatment interruption or de-escalation was not prospectively captured in a fraction of patients, the study does indicate the need to keep the interruptions/de-escalations to a minimum to avoid the increased risk of mortality, specifically in those patients not in remission. The strengths of the study include a large data set from one of the severely COVID-19 hit, low SDI regions [16]. Additionally, this study comprises broad hematological malignancies including leukemia, lymphoma, and myeloma, with a relatively longer follow-up post-COVID-19. As the study included patients from India up to March 20, 2021, most of the patients were unvaccinated, and therefore, the impact of vaccination in this population was not analysed. In conclusion, the study highlights the high mortality due to COVID-19 in patients with HM especially AML and post-HCT patients. More than one billion population in India is already vaccinated and it will be interesting to know the impact of the COVID-19 vaccine on the outcome of cancer patients in the future.

## Supplementary information

Supplement Table 1

Supplement Table 2

Supplement Table 3

Supplement Table 4

Supplement Figure 1

Supplement Figure 2

Supplement Figure 3
