# Supplementary material for: Outcomes of patients with hematologic malignancies and COVID-19 from the Hematologic Cancer Registry of India
Source: Blood Cancer J. 2022 Jan 5;12(1):2. doi: 10.1038/s41408-021-00599-w (PMC8728704; doi:10.1038/s41408-021-00599-w)
Supplement: Supplementary file 1 — Supplement Table 1 [file 41408_2021_599_MOESM1_ESM.docx]

**Supplement Tables**

**Table 1: Association of COVID-19 specific therapy with mortality in moderate/severe disease**

| **variables** | **Patient Status**  **(Moderate and Severe Covid-19 cases only)** | | | **P value** |
| --- | --- | --- | --- | --- |
|  | **Over all (186)** | **Alive (100)** | **Dead (86)** |  |
|  | **n (%)** | **n (%)** | **n (%)** |  |
| **Steroid** | 141 (75.81) | 78 (78.00) | 63 (73.26) | 0.451 |
| **Remdesivir** | 86 (46.49) | 47 (47.47) | 39 (45.35) | 0.772 |
| **Favipiravir** | 5 (2.70) | 4 (4.04) | 1 (1.16) | 0.374 |
| **HCQS** | 6 (3.24) | 4 (4.04) | 2 (2.33) | 0.687 |
| **Tocilizumab** | 14 (7.61) | 6 (6.06) | 8 (9.41) | 0.393 |
| **Prophylactic Anticoagulant** | 83 (44.86) | 48 (48.48) | 35 (40.70) | 0.288 |
| **Therapeutic anticoagulant** | 18 (9.73) | 9 (9.09) | 9 (10.47) | 0.753 |
| **Convalescent plasma** | 2 (1.08) | 0 (0.00) | 2 (2.33) | 0.215 |
| **HFNC** | 52 (28.11) | 22 (22.22) | 30 (34.88) | 0.056 |
| **Ventilatory support** | 66 (35.48) | 13 (13.00) | 53 (61.63) | <0.001 |
| **Either HFNC or Ventilatory support** | 86 (46.24) | 28 (28.00) | 58 (67.44) | <0.001 |
