# Supplementary material for: Outcomes of patients with hematologic malignancies and COVID-19 from the Hematologic Cancer Registry of India
Source: Blood Cancer J. 2022 Jan 5;12(1):2. doi: 10.1038/s41408-021-00599-w (PMC8728704; doi:10.1038/s41408-021-00599-w)
Supplement: Supplementary file 2 — Supplement Table 2 [file 41408_2021_599_MOESM2_ESM.docx]

**Table 2: Univariate and multivariable analysis of risk of severe COVID-19 among patients with Hematologic Malignancies**

| **Variables** | **Univariate Model for Moderate and Severe Covid-19** | | **Multivariable Model for Moderate and Severe Covid-19** | |
| --- | --- | --- | --- | --- |
|  | **OR (95% CI)** | **P Value** | **OR (95% CI)** | **P Value** |
| **Demographic factors** | | | | |
| **Age** |  |  |  |  |
| ≤20 | 1.00 |  | 1.00 |  |
| 21-40 | 2.54 (1.32 – 4.90) | **0.005** | 2.60 (1.31 – 5.15) | **0.006** |
| 41-60 | 3.51 (1.84 – 6.71) | **<0.001** | 3.44 (1.60 – 7.41) | **0.002** |
| ≥61 | 6.04 (3.01 – 12.10) | **<0.001** | 5.70 (2.43 – 13.35) | **<0.001** |
| **Diabetes** |  |  |  |  |
| Yes | 1.89 (1.18 – 3.04) | **0.009** | 1.20 (0.68 – 2.12) | 0.528 |
| No | 1.00 |  | 1.00 |  |
| **Hypertension** |  |  |  |  |
| Yes | 1.94 (1.17 – 3.19) | **0.010** | 1.09 (0.59 – 1.99) | 0.787 |
| No | 1.00 |  | 1.00 |  |
| **Hematologic Cancer related factors** | | | | |
| **ALL** | 1.00 |  | 1.00 |  |
| **AML** | 3.70 (2.06 – 6.67) | **<0.001** | 2.73 (1.45 – 5.12) | **0.002** |
| **Low Grade NHL** | 3.20 (1.68 – 6.09) | **<0.001** | 1.53 (0.71 – 3.26) | 0.275 |
| **High Grade NHL** | 1.55 (0.89 – 2.70) | 0.120 | 0.79 (0.42 – 1.52) | 0.485 |
| **Hodgkin Lymphoma** | 1.39 (0.55 – 3.49) | 0.483 | 0.84 (0.32 – 2.22) | 0.727 |
| **MM** | 2.88 (1.64 – 5.05) | **<0.001** | 1.15 (0.57 – 2.35) | 0.693 |
| **CML** | 1.73 (0.67 – 4.45) | 0.257 | 1.34 (0.49 – 3.68) | 0.574 |
| **Others** | 0.70 (0.17 – 2.86) | 0.624 | 0.38 (0.09 – 1.68) | 0.203 |
| **Cancer Treatment** | | | | |
| **Cancer to COVID Diagnosis Interval** |  |  |  |  |
| ≤6 Month | 1.00 |  |  |  |
| >6 Months | 1.07 (0.74 – 1.54) | 0.727 | - | - |
| **Malignancy Status** |  |  |  |  |
| Not in remission | 1.71 (1.12 – 2.60) | **0.013** | 1.85 (1.18 – 2.89) | **0.007** |
| Remission | 1.00 |  | 1.00 |  |
| **Systemic anti-cancer therapy** |  |  |  |  |
| Yes | 0.95 (0.63 – 1.43) | 0.811 | - | - |
| No | 1.00 |  |  |  |
| **Steroids (previous 4 weeks)** |  |  |  |  |
| Yes | 1.01 (0.70 – 1.44) | 0.975 | - | - |
| No |  |  |  |  |
| **Monoclonal Antibody (previous 4 weeks)*** |  |  |  |  |
| Yes | 0.77 (0.46 – 1.27) | 0.300 | - | - |
| No |  |  |  |  |
| **Post-Transplant** |  |  |  |  |
| Yes | 1.54 (0.70 – 3.37) | 0.280 | - | - |
| No |  |  |  |  |
| **COVID-19 related factors** | | | | |
| **ANC** |  |  |  |  |
| <0.5 | 1.17 (0.72 – 1.91) | 0.529 | - | - |
| ≥0.5 | 1.00 |  |  |  |
| **D Dimer (Baseline)** |  |  |  |  |
| <1000 | 1.00 |  |  |  |
| 1000 - 2000 | 0.86 (0.41 – 1.81) | 0.684 | - | - |
| >2000 | 1.56 (0.84 – 2.89) | 0.162 | - | - |
| **Ferritin (Baseline)** |  |  |  |  |
| < 500 | 1.00 |  | - | - |
| ≥ 500 | 2.25 (1.22 – 4.13) | **0.009** | - | **-** |
