# Supplementary material for: Outcomes of patients with hematologic malignancies and COVID-19 from the Hematologic Cancer Registry of India
Source: Blood Cancer J. 2022 Jan 5;12(1):2. doi: 10.1038/s41408-021-00599-w (PMC8728704; doi:10.1038/s41408-021-00599-w)
Supplement: Supplementary file 3 — Supplement Table 3 [file 41408_2021_599_MOESM3_ESM.docx]

**Table 3. Comparison of studies reporting the outcomes of COVID-19 in patients with hematological malignancies around the world.**

| Author, Country, Year | Study type, Sample size | Severity, median follow-up | Mortality | Factors affecting mortality | Factors NOT affecting mortality |
| --- | --- | --- | --- | --- | --- |
| Vijenthira et al,^5^  Asia-Europe-N.America, 2020 | Systematic review  3377 | 77% hospitalized,  NA | 34% in adults  4% in paediatrics  32% lymphoma/ CLL/ myeloma,  41% acute leukaemia | Age≥60,  hematologic malignancy  Non-white RR 2.2 | Recent anticancer Rx |
| Pagano et al,2020^6^ | EHA registry,3801 | 73% hospitalized | 31.2% mortality,40% mortality in AML and 42% in MDS | Age, active malignancy,AML associated with mortality. | CML,Obesity,ALL,NHL not associated with mortality in multivariable analysis |
| Wood et al,^8^ 2020 | ASH registry, 74 sites around world, 250 | 31% mild,  NA | 28% overall  42% mod-severe | Relapse/refractory disease  Estimated pre-COVID-19 prognosis <12 months |  |
| Passamonti et al,^9^  Italy,  2020 | Retrospective, multicenter,  536 | 84% hospitalized,  20 days | 37% | Old age HR 1.03  Progressive disease HR 2.1  AML HR 3.5,  Indolent NHL HR 2.19,  aggressive NHL HR 2.56,  Plasma cell dyscrasia HR 2.48  Severe COVID-19 HR 4.08 | Gender  comorbidity,  MDS/ MPN, ALL, CLPD, time since malignancy diagnosis, |
| Pinana et al,^10^  Spain,  2020 | Retrospective, multicentre,  367 | ~30% out-patient,  21-35 days | 31% in non-HCT patients  17/18% in auto/allo-HCT patients | Age>70 years OR 2.1  Uncontrolled disease OR2.9  ECOG 3-4 OR 2.56  ANC<0.5 OR 2.8  CRP>20 OR 3.3  Platelet<50 HR 3.6 | Gender, disease, HCT, chemo/ time since diagnosis  Antivirals (HCQ, lop/ritonavir, Remdesivir), steroids, Tocilizumab, ferritin, D-dimer, |
| Garcia-Suarez et al,^11^ Spain,  2020 | population-based registry,  833 | 62% severe-critical,  23% moderate  15% mild,  43 days | 33% | Age ≥60 HR 3.17  Comorbidity>2 HR 1.41  AML HR 2.22  MoAbs HR 2.02  Chemotherapy HR 1.5  HCQ HR 0.38 | Gender,  HCT,  Ph neg MPN  HMA  Steroids  Tocilizumab |
| Sharma et al,^15^  2021 | CIBMTR, 318 | 14% severe  24% moderate  49% mild,  21-25 days | 32% | Age>50 years HR 2.53  male sex HR 3.53  ≤12 months from HCT HR 2.67  Lymphoma > MM HR 2.41 | Race, ethnicity,  HCT-CI, IST within 6-months, |
| Ljungman et al,^16^ 2021 | EBMT registry,  382 | 32.2% allo-HCT & 38.4% auto-HCT required O2,  6-weeks | 28.4% | Age  PS  Need for ICU Immunodeficiency index | Underlying diagnosis, time from HCT, GVHD, ongoing IST |
| Current study | CHCRI,  565 | 30% moderate-severe disease, 76.5% hospitalized, 5-months | 19% whole cohort,  46% in moderate-severe COVID-19 | Age> 60 years HR 3.1  AML HR 2.92,  Post-HCT HR 4.11  HFNC/ ventilation HR 6.23  Malignancy Rx interruption/ de-escalation HR 2.93  ANC < 0.5 HR 1.6  d-dimer >2000 HR 3.33  ferritin ≥ 500 HR 3.43 | Gender,  Comorbidities  Lymphoma, myeloma, anticancer therapy, cancer-COVID-19 interval, CRP, ALC |
