# Supplementary material for: Outcomes of patients with hematologic malignancies and COVID-19 from the Hematologic Cancer Registry of India
Source: Blood Cancer J. 2022 Jan 5;12(1):2. doi: 10.1038/s41408-021-00599-w (PMC8728704; doi:10.1038/s41408-021-00599-w)
Supplement: Supplementary file 4 — Supplement Table 4 [file 41408_2021_599_MOESM4_ESM.docx]

**Table 4 COVID-19 Outcomes in various Hematologic Malignancies**

| **Variables** | **Total**  **(N=565)** | **Lymphoid** | | | | | **Myeloid** | |  |
| --- | --- | --- | --- | --- | --- | --- | --- | --- | --- |
|  |  | **ALL**  **(N=155)** | **High grade NHL (N=118)** | **Low grade**  **NHL**  **(N=57)** | **HL**  **(N=27)** | **MM**  **(N=93)** | **AML**  **(N=77)** | **CML**  **(N=23)** | **Others**  **(N=15)** |
| **Age** Mean(SD) | 40.84 (19.29) | 23.45 (13.46) | 46.18 (15.33) | 55.4 (15.83) | 36.07 (19.36) | 57.12 (9.78) | 37.23 (17.59) | 40.96 (13.96) | 49.33 (17.64) |
| **Comorbidity** | 147 (26.0) | 12 (7.74) | 36 (30.51) | 19 (33.33) | 9 (33.33) | 41 (44.09) | 22 (28.57) | 3 (13.04) | 5 (33.3) |
| **COVID severity** |  |  |  |  |  |  |  |  |  |
| Mild | 379 (67.1) | 123 (79.35) | 84 (71.19) | 31 (54.39) | 20 (74.07) | 53 (56.99) | 39 (50.65) | 16 (69.57) | 13 (86.7) |
| Moderate | 71 (12.6) | 12 (7.74) | 13 (11.02) | 11 (19.30) | 4 (14.81) | 16 (17.20) | 11 (14.29) | 3 (13.04) | 1 (6.7) |
| Severe | 115 (20.4) | 20 (12.90) | 21 (17.80) | 15 (26.32) | 3 (11.11) | 24 (25.81) | 27 (35.06) | 4 (17.39) | 1 (6.7) |
| **Malignancy Status – Remission** | 149 (27.0) | 49 (32.67) | 22 (18.97) | 17 (30.36) | 5 (18.52) | 16 (17.58) | 23 (30.26) | 13 (59.09) | 4 (28.6) |
| Non survivor **at Day7** | 37 (6.76) | 8 (5.19) | 5 (4.24) | 4 (7.02) | 1 (3.85) | 9 (9.78) | 9 (11.69) | 1 (4.35) | 0 (0.0) |
| Non survivor **at Day 14** | 60 (10.7) | 13 (8.4) | 8 (6.8) | 5 (8.8) | 1 (3.8) | 13 (14.3) | 16 (21.1) | 3 (13.0) | 1 (6.7) |
| Non survivor **at 1 month** | 81 (14.6) | 17 (11.1) | 15 (12.7) | 7 (12.5) | 2 (7.7) | 15 (16.9) | 20 (27.0) | 3 (13.0) | 2 (13.3) |
| Non survivor **at 3 months** | 111 (21.9) | 21 (14.5) | 20 (18.5) | 11 (22.9) | 3 (12.5) | 20 (25.6) | 30 (44.1) | 3 (13.6) | 3 (21.4) |
| Non survivor **at 6 months** | 116 (30.2) | 23 (21.1) | 20 (23.8) | 12 (33.3) | 3 (16.7) | 20 (36.4) | 32 (57.1) | 3 (18.8) | 3 (30.0) |
| **Anti-cancer**  **therapy** Interruption/  De-escalation | 398 (70.7) | 109 (70.3) | 93 (80.2) | 35 (61.4) | 21 (77.8) | 78 (83.9) | 50 (64.9) | 4 (17.4) | 8 (53.3) |
