# Supplementary material for: Outcomes of patients with hematologic malignancies and COVID-19 from the Hematologic Cancer Registry of India
Source: Blood Cancer J. 2022 Jan 5;12(1):2. doi: 10.1038/s41408-021-00599-w (PMC8728704; doi:10.1038/s41408-021-00599-w)
Supplement: Supplementary file 5 — Supplement Figure 1 [file 41408_2021_599_MOESM5_ESM.docx]

**Figure 1 Consort diagram**

Patients enrolled in the study

**n=588**

Excluded **(n=23)**

Non hematologic cancer /Incomplete outcome data details /diagnosis/Discrepant

(n=379)Mild COVID-19

**(n=71)**Moderate COVID-19

**(n=115)**Severe COVID-19

9n=

Severe COVID-19

Cases

**(n=263)** Hospitalized

**(n=109)** Not hospitalized

**(n=2)** Advised hospitalization but refused

**5** - Missing

**(n=166)** Hospitalized

**(n=17)** Not hospitalized

**(n=2)** Advised hospitalization but refused

**(n=1)** Missing

**(n=33)** denied ICU admission in favor of palliation

Patients included in the analysis **(n=565)**

**Figure 2: Point wise mapping of cases enrolled in CHCRI Study**
