# Supplementary figures and images for: Outcomes of patients with hematologic malignancies and COVID-19 from the Hematologic Cancer Registry of India
Source: Blood Cancer J. 2022 Jan 5;12(1):2. doi: 10.1038/s41408-021-00599-w (PMC8728704; doi:10.1038/s41408-021-00599-w)

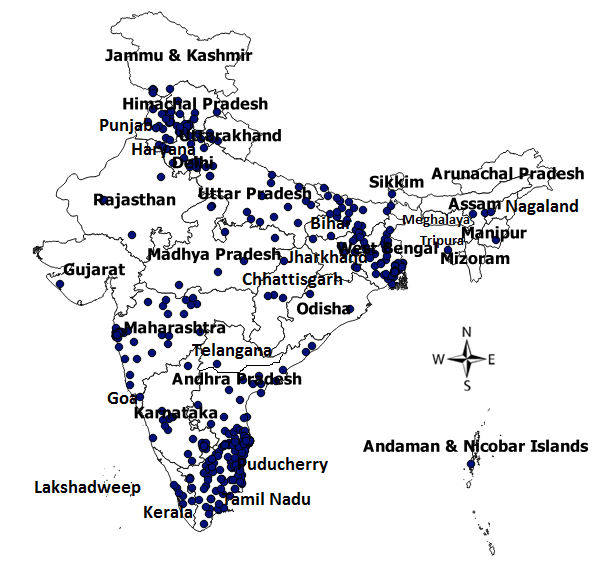


**Figure 2 Point wise mapping of cases**

Supplement: Supplementary file 6 — Supplement Figure 2 [file 41408_2021_599_MOESM6_ESM.docx]
