# Supplementary material for: Outcomes of patients with hematologic malignancies and COVID-19 from the Hematologic Cancer Registry of India
Source: Blood Cancer J. 2022 Jan 5;12(1):2. doi: 10.1038/s41408-021-00599-w (PMC8728704; doi:10.1038/s41408-021-00599-w)
Supplement: Supplementary file 7 — Supplement Figure 3 [file 41408_2021_599_MOESM7_ESM.docx]

**Figure 3 Overall Survival of COVID-19 patients with Hematologic Malignancy with respect to**

**a) Malignancy Subtype b) Anti-Cancer Treatment interruption/de-escalation**

**
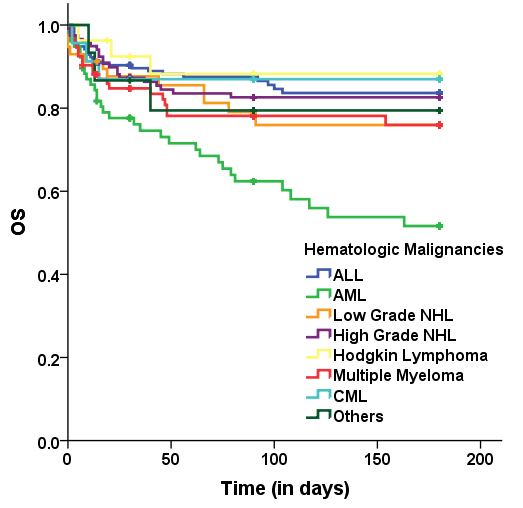

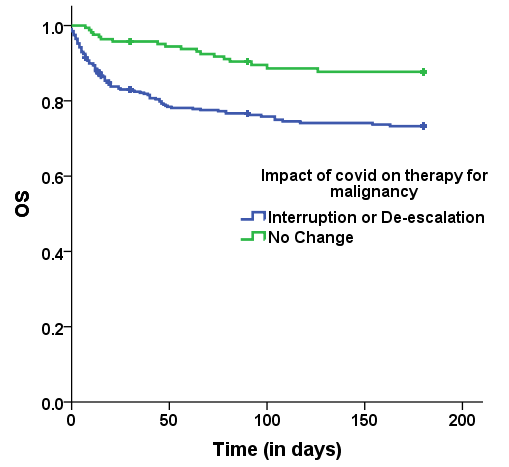
**

b)

a)
